# Chemical and Thermal Unfolding of a Global Staphylococcal Virulence Regulator with a Flexible C-Terminal End

**DOI:** 10.1371/journal.pone.0122168

**Published:** 2015-03-30

**Authors:** Avisek Mahapa, Sukhendu Mandal, Anindya Biswas, Biswanath Jana, Soumitra Polley, Subrata Sau, Keya Sau

**Affiliations:** 1 Department of Biotechnology, Haldia Institute of Technology, Haldia, West Bengal, India; 2 Department of Biochemistry, Bose Institute, Kolkata, West Bengal, India; Instituto Tecnologia Quimica e Biologica; Universidade Nova de Lisboa, PORTUGAL

## Abstract

SarA, a *Staphylococcus aureus*-specific dimeric protein, modulates the expression of numerous proteins including various virulence factors. Interestingly, *S*. *aureus* synthesizes multiple SarA paralogs seemingly for optimizing the expression of its virulence factors. To understand the domain structure/flexibility and the folding/unfolding mechanism of the SarA protein family, we have studied a recombinant SarA (designated rSarA) using various *in vitro* probes. Limited proteolysis of rSarA and the subsequent analysis of the resulting protein fragments suggested it to be a single-domain protein with a long, flexible C-terminal end. rSarA was unfolded by different mechanisms in the presence of different chemical and physical denaturants. While urea-induced unfolding of rSarA occurred successively via the formation of a dimeric and a monomeric intermediate, GdnCl-induced unfolding of this protein proceeded through the production of two dimeric intermediates. The surface hydrophobicity and the structures of the intermediates were not identical and also differed significantly from those of native rSarA. Of the intermediates, the GdnCl-generated intermediates not only possessed a molten globule-like structure but also exhibited resistance to dissociation during their unfolding. Compared to the native rSarA, the intermediate that was originated at lower GdnCl concentration carried a compact shape, whereas, other intermediates owned a swelled shape. The chemical-induced unfolding, unlike thermal unfolding of rSarA, was completely reversible in nature.

## Introduction

*Staphylococcus aureus*, a disease-causing Gram-positive bacterium, produces many virulence factors (e.g., protein A, clumping factor, fibronectin-binding protein, capsule, hemolysins, biofilm, phenol-soluble modulins, etc.) for its successful colonization and infection [1]. Several regulators (such as *agr*, *saeRS*, *codY*, *sigB*, *sarA*, etc.) control the expression of virulence factors in *S*. *aureus* [2–5]. Most times, virulence regulators act upon each other to optimize the expression of the virulence factors [3]. Of the virulence regulators, *agr* and *sarA*, were considered as the global virulence regulators as they independently or co-dependently control the synthesis of most virulence factors in *S*. *aureus* [4–7].

SarA, a protein product of *sarA*, modulates the transcription of nearly 120 genes (including various virulence factor-encoding genes) in *S*. *aureus*. In addition, it regulates its synthesis as well as the production of RNAIII transcript from the *agr* locus [4–7]. Apparently, SarA controls the transcription of the genes by binding to their respective promoters. The DNA binding activity of SarA was demonstrated to be influenced by its phosphorylation-dephosphorylation status [8, 9]. The redox state and pH of the buffer were also reported to have effects on its DNA binding affinity [10]. Interestingly, SarA also regulates the gene expression at the post-transcriptional level as its binding to various mRNA species altered their stability and turnover [11]. SarA is highly abundant in *S*. *aureus* and even showed binding to the *att* site of phage λ [10]. In solution, this global regulator exists as a dimer and is predominantly α-helical [12]. The X-ray crystal structure of SarA revealed it to be a winged-helix DNA binding protein harbouring two globular monomers [13]. Each SarA monomer displays multiple α-helices, β-strands and loops. The putative DNA binding region of SarA is composed of a helix-turn-helix (HTH) motif and a hairpin, which possibly bind to the major and minor grooves of DNA, respectively. Dimerization of SarA occurs by an N-terminal end α-helix that is not involved in the HTH motif formation.

The genome of *S*. *aureus* N315 carries genes for encoding as many as 11 SarA paralogs, namely, SarA, MgrA, Rot, SarR, SarS, SarT, SarU, SarV, SarX, SarY, and SarZ [14]. Of the SarA paralogs, SarA, SarR, SarS, SarZ, MgrA, and Rot are expressed efficiently by several laboratory and clinical strains of *S*. *aureus* [15]. In depth studies have indicated that SarA paralogs, together or individually, control the expression of numerous genes including those involved in pathogenesis, autolysis, antibiotic resistance, etc. [6, 7, 14]. Like SarA, SarR, SarS, SarZ, Rot, and MgrA also possess a winged-helix conformation [14, 16–19]. Some of the Sar family members (namely, SarA, SarX, and SarZ) are also expressed by *S*. *epidermidis*, another staphylococcal pathogen [6]. Under the context of evolution and propagation of multiple antibiotic-resistant *S*. *aureus* strains worldwide, SarA and other family members, due to their pivotal role in the pathogenesis, were considered as one of the promising drug targets [7, 20]. A few screened (small) molecules, which inhibited the interaction between MgrA and the cognate DNA, indeed yielded encouraging results in the laboratory [20, 21].

A nascent polypeptide becomes biologically active once it is folded properly in the cell. To understand the protein folding mechanism, synthesis of the folding intermediate (if any), and the conformational stability, unfolding of numerous proteins have been investigated in the last ~40 years using one or more denaturants (e.g., urea, guanidine hydrochloride, heat, etc.) and sensitive probes [22–29]. Apparently, proteins are unfolded either by a two-state mechanism (with the formation of no intermediate) or by a three- or higher-state mechanism via the synthesis of one or more intermediates. In addition to providing clues on the folding mechanism and stability of proteins, unfolding data are also found to be useful in diverse biotechnological fields including drug discovery [30–35].

The C-terminal halves of SarA and the related proteins are composed of the amino acid residues with comparatively higher crystallographic *B*-values (or temperature factors) [13, 16–19]. As the higher crystallographic *B*-value indicates the increased flexibility in protein [36, 37], the C-terminal ends of Sar proteins may be more flexible than their N-terminal ends. The C-terminal ends of most Sar proteins also appeared to possess higher fraction of surface-accessible amino acid residues. Thus far, no biochemical study has been carried out to verify the flexibility/domain structure of Sar proteins and their surface-exposed residues. In addition, little is known about the folding-unfolding mechanisms of SarA and the homologous proteins though such data could be exploited in the anti-staphylococcal drug discovery. In the present study, we have used a recombinant SarA (rSarA) as a representative of the SarA protein family and demonstrated that it is a single domain protein with a flexible C-terminal end. Both the urea- and GdnCl-induced unfolding of rSarA occurred via the generation of two intermediates. The rSarA intermediates generated specifically by GdnCl appeared to possess a molten globule-like structure [38] and were dimeric in nature. While the chemical-induced unfolding of rSarA was reversible, thermal unfolding of rSarA was irreversible in nature.

## Materials and Methods

### Materials

Glutaraldehyde, guanidine hydrochloride (GdnCl), acrylamide, 8-anilino-1-napthalenesulfonate (ANS), bis-acrylamide, phenylmethane sulfonylfluoride (PMSF), isopropyl-β-D-1-thiogalactopyranoside (IPTG), urea, and different proteolytic enzymes (proteinase K, endoproteinase AspN, and chymotrypsin) were purchased from Sigma, Merck or SRL. All other materials were of the maximum purity available. Markers, restriction and modifying enzymes, the polymerase chain reaction (PCR) kit, gel extraction kit, plasmid DNA purification kit, Ni-NTA resin, and oligonucleotides were procured from Qiagen, Fermentas, GE Healthcare Biosciences Ltd., Genetix Biotech Asia Pvt Ltd., and Hysel India Pvt Ltd. The alkaline phosphatase-tagged goat anti-mouse antibody (IgG1-AP) and anti-His antibody were bought from Santa Cruz Biotechnology Inc. Radioactive [γ-^32^P] ATP was purchased from the Bhabha Atomic Research Center. *S*. *aureus* strain Newman was gifted by Prof. C. Y. Lee (the University of Arkansas for Medical Sciences). *E*. *coli* BL21(DE3) and plasmid pET28a were donated by the late Prof. P. Roy (Bose Institute). Oligonucleotides SarA1 (5’ CATACCATGGC AATTACAAAAATCAATG 3’), SarA2 (5’CATACTCGAGTAGTTCAATTTCGTTGT TTG3’), Hla1 (5’Acatagctaattttattg 3’), and Hla2 (5’ctattagatattt ctatg 3’) were used in the present study.

### Growth of bacteria

All of the *E*. *coli* strains including *E*. *coli* XL1-Blue and *E*. *coli* BL21 (DE3) were cultivated in Luria-Bertani broth [39]. *S*. *aureus* Newman was routinely grown in Trypticase Soy broth [40]. Growth media were supplemented with appropriate antibiotics when necessary.

### Molecular biological techniques

All of the molecular biological methods such as polymerase chain reaction (PCR), labelling of DNA fragment by radioactive [γ-^32^P] ATP, plasmid DNA isolation, restriction enzyme digestion, agarose gel electrophoresis, ligation, competent *E*. *coli* cell preparation, DNA transformation, protein estimation, native polyacrylamide gel electrophoresis, SDS-PAGE, native-PAGE, staining of polyacrylamide gel, Western blotting, isolation of chromosomal DNA from *S*. *aureus* Newman, sequencing of the PCR-made DNA fragments, and gel shift assay were performed as reported earlier [39–44].

### Purification of rSarA

To purify rSarA (a C-terminally histidine-tagged SarA), a DNA fragment was produced (by PCR using primers SarA1 and SarA2 and the *S*. *aureus* Newman genomic DNA as template) and digested with *Nco*I and *Xho*I followed by the ligation of the resulting fragment into the identical sites of pET28a, an *E*. *coli*-specific expression vector. The ligated DNAs were transformed into *E*. *coli* XL1 Blue. Plasmids isolated from 3–4 transformants were found to possess the DNA insert of the right size. A plasmid with the correct DNA insert (confirmed by DNA sequencing) was stocked and designated as p1311. An *E*. *coli* strain (namely, SAU1311) was constructed by transforming p1311 to *E*. *coli* BL21(DE3). The cloning attached eight additional residues (including six histidine residues) at the C-terminal end of SarA (UniProt Code: P0C1U6). Finally, rSarA was purified from SAU1311 extract by Ni-NTA affinity chromatography as described earlier [44]. The eluted rSarA was dialyzed against buffer A [20 mM phosphate buffer (pH 8.0), 100 mM NaCl, and 5% glycerol] for 12–16 h at 4°C. An SDS-13.5% PAGE analysis revealed that the elution fraction primarily carried rSarA (S1A Fig), which expectedly reacted with the anti-His antibody (S1B Fig). The molar concentration of rSarA was determined using the theoretical mass (determined from the rSarA sequence with a software program) of its monomer and the amount of rSarA content in buffer A. To check as to whether rSarA is functional, a gel shift assay was carried out using 0.05–3 μM rSarA and ^32^P-labeled hla DNA (prepared using Newman chromosomal DNA and primers Hla1 and Hla2). The results showed the appreciable binding of rSarA to hla DNA (S1C Fig). The amounts of hla DNA bound by different concentrations of rSarA were determined using the scanned band intensity data from several similar autoradiograms (including the autoradiogram shown in S1C Fig). The non-linear fitting of the scanned data were performed using a standard procedure as described previously [40, 44]. The yielded (apparent) equilibrium constant (i.e., the concentration of rSarA required for half-maximal binding) was found to be 286±11 nM (S1D Fig). The data together suggest that rSarA purified from a heterologous system is quite active and possesses a proper conformation.

### Limited proteolysis

To map the flexible regions in rSarA, limited proteolysis of this protein was carried out individually with proteinase K, chymotrypsin and endoproteinase AspN essentially by a procedure as reported earlier [43]. To identify the proteolytic cut sites, N-terminal ends of some rSarA fragments were sequenced as described [43]. To determine the molecular masses of the proteolytic fragments (yielded from 4 h of digestion), their MALDI-TOF analysis was performed as stated before [43].

### Spectroscopic investigation

To know about the secondary and tertiary structures of rSarA, far-UV Circular Dichroism (CD) spectrum (200–260 nm) and near-UV CD spectrum (250–310 nm) of this protein were recorded as described [43, 45, 46]. rSarA concentrations for near- and far-UV CD experiments were 30 and 10 μM, respectively.

rSarA carries no Trp residue but carries six Tyr residues at positions 18, 42, 51, 52, 62, and 79. To determine the tertiary structure of rSarA, the intrinsic Tyr fluorescence spectrum (λ_ex_ = 280 nm and λ_em_ = 290–340 nm) of this macromolecule (10 μM) was also recorded as described [43, 45–47].

To obtain clues about the presence of the hydrophobic surface in rSarA, fluorescence spectrum (λ_ex_ = 360 nm and λ_em_ = 400–600 nm) of ANS (100 μM) bound to this protein (10 μM) was recorded as demonstrated [45–47]. To identify the aggregation of rSarA at 25°-85°C, light scattering of the macromolecule (10 μM) at 360 nm was monitored as reported [43].

### Denaturation and renaturation of rSarA

To understand the chemical-induced denaturation of rSarA, aliquots (each 10 μM) of this protein were incubated with 0–5 M GdnCl or 0–7 M urea separately for 16–18 h at 4°C followed by the recording of their far-UV CD, near-UV CD, Tyr fluorescence and ANS fluorescence spectra as illustrated above. To unfold rSarA, the freshly made solution of GdnCl or urea was always used. The *C*_m_ (concentration of denaturant at the midpoint of unfolding transition) and the *f*_u_ (fraction of unfolded rSarA molecules) values were determined by the standard methods [25, 43] using the above spectroscopic data. Urea-induced unfolding of rSarA (40 μM) in buffer A was also investigated by transverse urea gradient gel electrophoresis (TUGE) as described [45, 48]. Refolding of the GdnCl-denatured rSarA was investigated by CD spectroscopy as depicted earlier [43]. To see as to whether the refolded rSarA has regained any DNA binding activity, gel shift assay was performed as described above.

To investigate the thermal unfolding of rSarA, aliquots (each 10 μM) of this protein were incubated for 3–5 min at 25°-80°C followed by the recording of their far-UV CD spectra at the respective temperature by a standard procedure [45,46].

To observe as to whether thermal unfolding is reversible in nature, temperature of rSarA (10 μM) in buffer A was increased steadily from 25° to 80°C prior to its cooling down slowly to 25°C. Buffer A without the protein was treated similarly. Finally, the far-UV CD spectra of the cooled buffer A and rSarA were recorded as stated above. The CD signal of the former was deducted from that of the latter. The far-UV CD spectra of native and unfolded rSarA (each 10 μM) were also recorded to assess the refolding.

### Understanding shape and size of rSarA

To learn as to whether rSarA (10 μM) forms dimer in solution at 0–5 M GdnCl/0-7 M urea, a glutaraldehyde-mediated cross-linking experiment was carried out as reported earlier [43, 44]. To know about the shape and size of rSarA (20 μM) in solution at 0–5 M GdnCl/0-7 M urea, we performed analytical gel filtration chromatography as demonstrated previously [43, 44]. To determine the hydrodynamic radius of rSarA in the presence/absence of GdnCl, dynamic light scattering (DLS) experiment of rSarA (50 μM) was carried out essentially as reported earlier [49].

### Statistical analysis

All of the results were provided here as the means of at least three separate experiments with the standard deviation. Mean and standard deviation were estimated using Microsoft Excel.

## Results

### Flexible regions in rSarA

**Fig 1:**
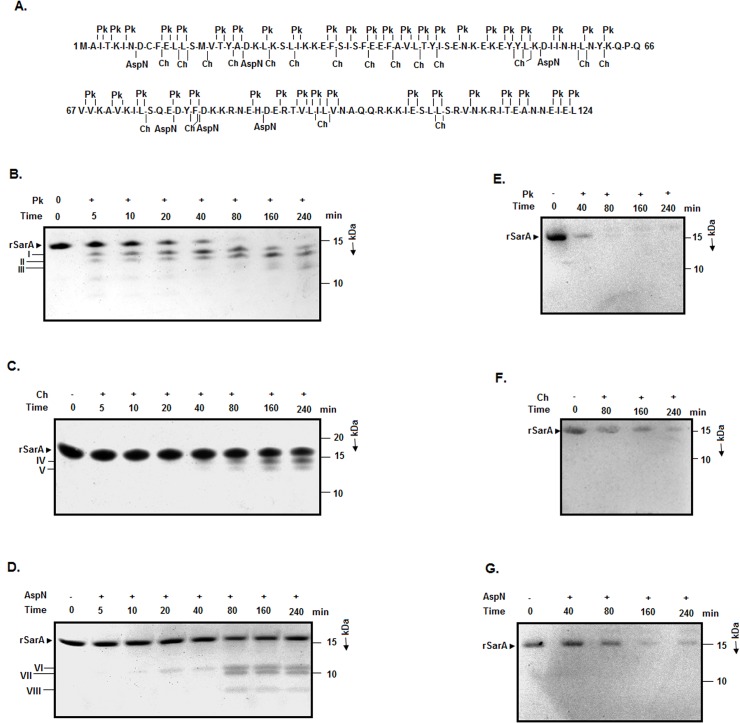
Limited proteolysis of rSarA. (A) Amino acid sequence of SarA with the cleavage sites of proteinase K (Pk), chymotrypsin (Ch), and endoproteinase AspN (AspN). The cut sites of each enzyme in the SarA sequence were identified using the ‘PeptideCutter’ program in ExPasy server. Analyses of the proteinase K (B)-, chymotrypsin (C)-, and endoproteinase AspN (D)-cleaved rSarA fragments by SDS-13.5% PAGE. I–VIII indicates the major proteolytic fragments of rSarA. The rSarA-specific bands are denoted by an arrowhead. Western blotting analyses of the Pk (E)-, Ch (F)-, and AspN (G)-digested rSarA fragments using an anti-His antibody. The molecular masses (in kDa) of marker proteins are presented at the right side of the pictures.

Limited proteolysis was used widely to identify the domain structure and the flexible region in proteins [27, 43]. Our bioinformatic analyses revealed that SarA possessed more than five cleavage sites for various proteolytic enzymes including endoproteinase AspN, chymotrypsin, and proteinase K (Fig 1A and data not shown). If SarA is truly a single-domain protein with no flexible region, it will show little sensitivity to the proteolytic enzyme. To verify this hypothesis, we performed limited proteolysis of rSarA with endoproteinase AspN, chymotrypsin, and proteinase K separately. Fig 1B shows the production of two fragments (designated I and II) at the early stage of digestion of rSarA with proteinase K. Both fragments were, however, degraded gradually upon prolonged digestion with proteinase K. At the late stage of digestion, proteinase K generated fragment III from rSarA, which apparently possesses 2 kDa less mass than that of fragments I and II. Cleavage of rSarA with chymotrypsin (Fig 1C) also resulted in two fragments (IV and V) (with the molecular masses ranging from ~14–13.5 kDa) but comparatively at the late stage. Contrary to above, proteolysis of rSarA with endoproteinase AspN at the early stage primarily yielded one fragment (VI) with the molecular mass of ~11 kDa (Fig 1D). Two smaller fragments (VII and VIII) were generated from the endoproteinase AspN digestion of rSarA at the relatively late stage. No proteolytic fragment interacted with the anti-His antibody though it reacted with rSarA as expected (Fig 1E–1G). The data indicate that proteinase K has removed the polyhistidine tag of rSarA as it possesses several cut sites at the C-terminal end of this protein (Fig 1A and data not shown). Similarly, fragments IV and V might have been generated by the chymotrypsin-catalyzed cleavage of the peptide bond(s) linked to the Leu/His residue at the C-terminal end of rSarA. Conversely, fragment VI must have been generated by the endoproteinase AspN-mediated digestion of the peptide bond formed with the Asp residue at position 88 of rSarA (Fig 1A).

To accurately map the cleavage sites in rSarA, the molecular masses of the proteolytic fragments (I-VIII) were determined by MALDI-TOF analysis (see Materials and Methods for details). Table 1 show that the masses of the fragments vary from 14698.98 to 6300.50 Da. The experimental masses of the rSarA fragments were compared with the theoretical masses of the rSarA fragments produced by random digestion. The results suggest that the fragments I, II, III, IV, V, VI, VII, and VIII are made of the residues A2-L125, S14-L125, T17-T116, A2-L125, A2-L108, A2-H87, A2-F80, and A2-K54, respectively. Additional sequencing analysis showed that the five N-terminal end residues of fragments I, II, and IV are AITKI, SMVTY, and AITKI, respectively (Table 1). Taken together, a total of four peptide bonds (formed by the amino acid residues Leu 13 and Ser 14, His 87 and Asp 88, Leu 108 and Ser 109, Leu 125 and Glu 126) of rSarA were digested by three proteolytic enzymes used in the study. Of the sensitive peptide bonds, the bond generated by Leu 125 and Glu 126 is in the polyhistidine tag of rSarA (data not shown).

**Table 1:** Analyses of different protease-cleaved fragments.

Enzyme	rSarA fragments	N-terminal end amino acids of rSarA fragment^A^	Mass of rSarA fragments^B^ (Da)	Probable region in the rSarA fragments^C^
Proteinase K	I	AITKI	14698.98	A2-L125
Proteinase K	II	SMVTY	13338.54	S14-L125
Proteinase K	III	-	12000.87	T17-T116
Chymotrypsin	IV	AITKI	14692.25	A2-L125
Chymotrypsin	V	-	12744.23	A2-L108
Endoproteinase AspN	VI	-	10268.44	A2-H87
Endoproteinase AspN	VII	-	9359.76	A2-F80
Endoproteinase AspN	VIII	-	6300.50	A2-K54

^A^N-terminal end residues of rSarA fragments I, II, and IV were determined by sequencing.

^B^Molecular masses of the rSarA fragments were determined by MALDI-TOF analysis.

^C^The masses of the designated regions of SarA (determined by ProtParam on ExPasy Server) were identical/very close to those estimated by MALDI-TOF analysis.

### Urea-induced unfolding of rSarA

**Fig 2:**
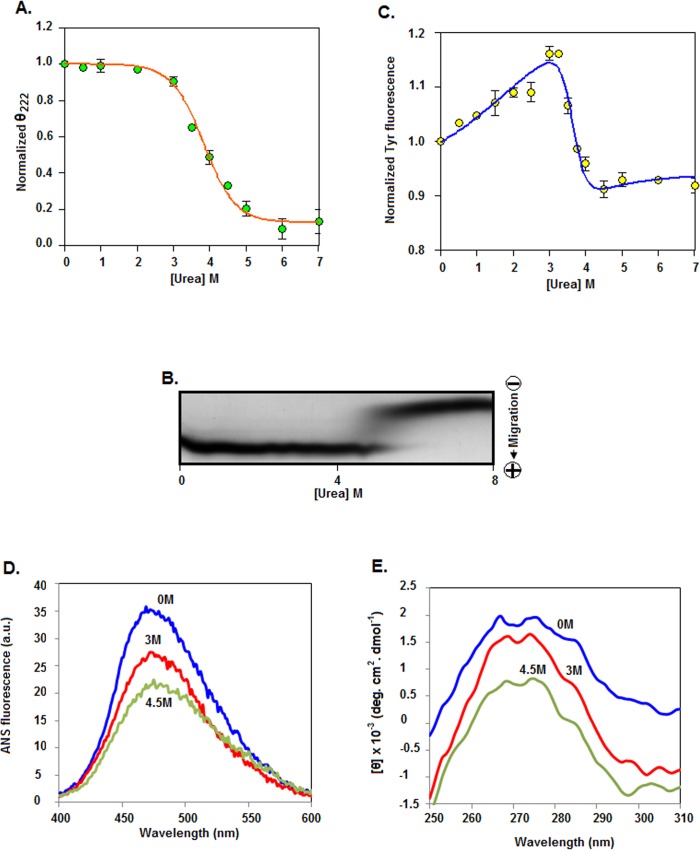
Urea-induced unfolding of rSarA. (A) The θ_222_ (ellipticity at 222 nm) values, derived from the far-UV CD spectra of 0-7M urea-equilibrated rSarA (S2A Fig), were normalized [43] and plotted against the correspnding urea concentrations. (B) Transverse urea-gradient gel electrophoresis of native rSarA. (C) The Tyr fluorescence intensity values (at 308 nm), extracted from the Tyr fluorescence spectra of 0–7 M urea-equilibrated rSarA (S2B Fig), were plotted as described above. The line through the Tyr fluorescence or CD values denotes the best-fit curve. ANS fluorescence (D) and near-UV CD (E) spectra of rSarA at 0, 3, and 4.5 M urea.

To gather information about the folding/unfolding mechanism of SarA, we investigated the urea-induced unfolding of rSarA by various *in vitro* techniques including far-UV CD (S2A Fig), and intrinsic Tyr fluorescence spectroscopy (S2B Fig). The ellipticity values of rSarA (at 222 nm) when plotted against the corresponding urea concentrations yielded a curve with the sigmoidal shape (Fig 2A). The passage of rSarA across a 0–8 M urea gradient gel also produced a monophasic curve-like protein band with an extremely faded transition region (Fig 2B). In contrast, a biphasic curve was observed when Tyr fluorescence intensity values of rSarA (at 308 nm) were plotted against the matching urea concentrations (Fig 2C). There was nearly 16% increase of the Tyr fluorescence intensity when the urea concentrations were enhanced from ~0 to 3 M. Upon further increasing the urea concentrations to 4.5 M, it was decreased about 26%. The fluorescence intensity values were, however, notably unaltered at ~5–7 M urea. As expected for Tyr fluorescence, no substantial change in the λ_max_ (wavelength of emission maxima) values was observed at 0–7 M urea. The drastic reduction of CD or Tyr fluorescence signals at ~3–4.5 M urea, however, indicates the initiation of unfolding (as well as the loss of structure) of rSarA at the urea concentration of >3 M. The unfolding of rSarA was completed mostly at ~4.5 M urea. rSarA existed solely as an unfolded form at ~5–7 M urea. Analysis of the three-dimensional structure of SarA [13] by Swiss-Pdb Viewer (at default setting) revealed that one (Tyr52) of its six Tyr residues remained exposed to its surface (data not shown). It is not clear as to whether the increase or decrease of Tyr fluorescence intensity of rSarA at different urea concentrations occurred due to burial or exposure of all or some of its Tyr residues.

To check the reversibility of the urea-induced unfolding of rSarA, the denatured form of this protein was analyzed by TUGE. As observed with the native rSarA (Fig 2B), migration of unfolded rSarA across a urea-gradient gel also resulted in a monophasic curve-like protein band with a pale transition region (S3A Fig). Such data indicate the complete reversibility of the urea-induced unfolding of rSarA though the unfolding/refolding reaction seemed to be slow. Additional studies by the far-UV CD spectroscopy (S3B Fig), and gel shift assay (S3C Fig) revealed that refolding of the unfolded rSarA also restored the secondary structure and the DNA binding activity of rSarA almost completely.

The curves, generated by plotting the fraction of unfolded rSarA molecules (determined using the spectroscopic data from Fig 2A and 2C) against the related urea concentrations, did not coincide with each other (data not shown). The CD data of rSarA fit best with the two-state model [25] that yielded the *C*_m_ value of 3.83±0.06 M. Conversely, the Tyr fluorescence data fit best with the three-state model [24, 25] that resulted in the *C*_m_ values of 1.74±0.09 and 3.64±0.05 M. The *C*_m_ value of 5.13±0.21 M was derived by analyzing the TUGE data with the two-state model. Taken together, the urea-induced unfolding of rSarA might have been proceeded through three steps with the formation of two intermediates. While the first intermediate might be generated at ~0–3 M urea, the second intermediate could be formed at ~3–4.5 M urea. The rSarA intermediates seemed most populated at ~3 and ~4.5 M urea, as the unfolding curves (particularly that generated using Tyr fluorescence intensity values) showed small plateau regions at these urea concentrations. To determine the properties of the predicted rSarA intermediates, both the ANS fluorescence and near-UV CD spectra of rSarA were recorded in the presence of 0, 3, and 4.5 M urea. The ANS fluorescence intensity (at 480 nm) of 3 or 4 M urea-equilibrated rSarA was less than that of this protein at 0 M urea, indicating the presence of a comparatively less hydrophobic surface area in both the intermediates (Fig 2D). The rSarA intermediates, as evident from the near-UV CD spectroscopy, however, contain a substantial extent of tertiary structure at 3 or 4.5 M urea (Fig 2E), suggesting that these intermediates are not a molten globule [38].

### Effects of urea on rSarA dimer

**Fig 3:**
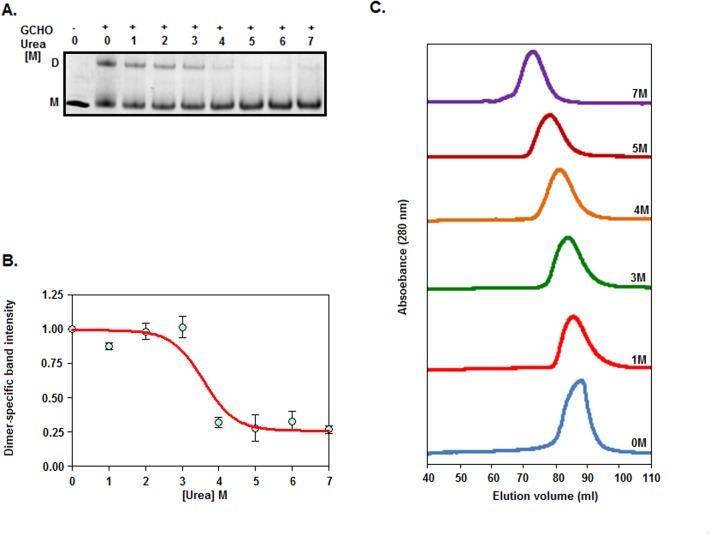
Effects of urea on the size and shape of rSarA. (A) Glutaraldehyde (GCHO)-mediated crosslinking of rSarA at 0–7 M urea. The crosslinked rSarA molecules were analyzed by SDS-13.5% PAGE. D and M indicate dimeric and monomeric rSarA, respectively. (B) The dimer-specific band intensity values were determined (from Fig 3A) and plotted versus the related urea concentrations as described [43]. (C) Gel filtration chromatograms of rSarA at the indicated concentrations of urea.

To determine as to whether the dissociation of dimeric rSarA takes place prior to the initiation of its unfolding with urea, we carried out a glutaraldehyde-mediated crosslinking experiment of rSarA in the presence of 0–7 M urea. Fig 3A shows that the intensities of the dimeric-specific rSarA bands at 0–3 M are higher than those at 4–7 M urea. Fig 3B shows the plot of dimer-specific band intensity versus the matching urea concentrations. There was reduction of dimer-specific band intensity from ~100 to 30% upon increasing the urea concentrations from 3 to 4 M. The midpoint of the yielded curve appears to be 3.58±0.23 M, suggesting a near coupled unfolding and dissociation of rSarA at 0–7 M urea. To verify the proposal, the gel filtration chromatography of rSarA was performed in the presence of 0–7 M urea. All of the samples produced a single peak with a distinct elution volume (Fig 3C). The retention volumes of 0, 1, 3, 4, 5, and 7 M urea-equilibrated rSarA were 88.44, 86.23, 84.15, 81.55, 78.63, and 73.27 ml, respectively, indicating that all of the urea-treated rSarA were eluted prior to the urea-untreated rSarA. Using the elution volumes of urea-untreated rSarA and some monomeric proteins (data not presented), the apparent molecular mass of native rSarA was found to be 31.95 kDa, indicating that it exists primarily as a dimer in the aqueous solution. Presently, the apparent molecular mass of any urea-treated rSarA is not clear. The elution profiles of rSarA, however, provided valuable clues about its shape in the presence of urea. An earlier elution of rSarA at 5 or 7 M urea might be due to the near completely or entirely complete unfolded form of this protein under these conditions. At 1–3 M urea, swelling of dimeric rSarA possibly led its elution prior to the elution of the native rSarA. rSarA intermediate formed at ~3 M urea (described above) could primarily be a dimeric rSarA with a swelled conformation. Most of the rSarA homodimers at 4 M urea might have been dissociated to rSarA monomers. If the two rSarA forms are stable enough, they should be eluted separately. The calculated elution volume of rSarA monomer (in the absence of urea) was 95.25 ml. The rSarA in the presence of 4 M urea, however, did not yield any peak with the retention volume corresponding closely to that of rSarA monomer. As the elution volume of rSarA at 4 M urea was about 8 ml higher than that of this protein at 7 M urea, there was no unfolding of monomeric form. The rSarA monomers were also not aggregated as no peak appeared at the void volume (42.38 ml) region. Taken together, the single peak generated from the 4 M urea-treated rSarA indicates an average of its two forms, which might be in equilibrium with each other. Previously, several other dimeric proteins, which swelled in the pre-transition region and dissociated in the transition region, showed a similar united peak in the transition region [43, 50, 51].

### GdnCl-induced unfolding of rSarA

**Fig 4:**
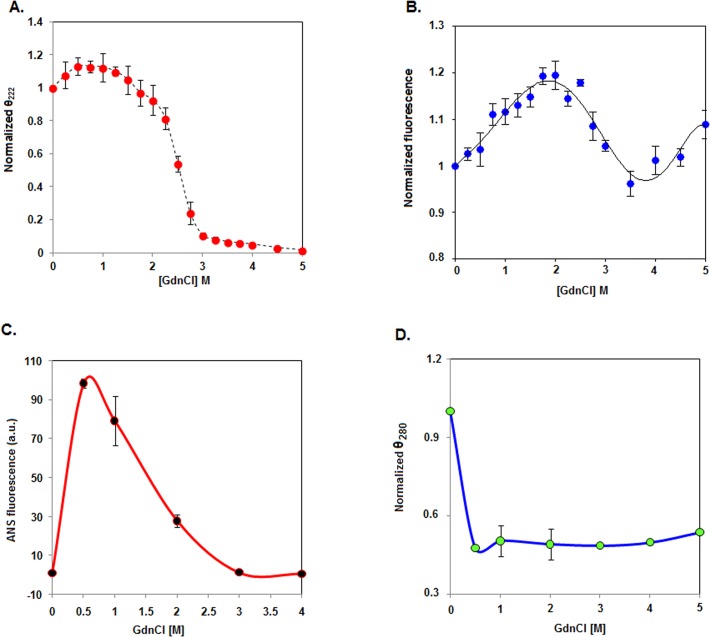
Unfolding of rSarA by GdnCl. (A) The plot of normalized θ_222_ (ellipticity at 222 nm) values versus the GdnCl concentrations was constructed using the recorded CD data in S4A Fig (B) The intrinsic Tyr fluorescence intensity values of rSarA at 306 nm (extracted from S4B Fig) were plotted against the corresponding GdnCl concentrations. The line through the fluorescence values indicates the best-fit curve. (C) ANS fluorescence intensity values of rSarA (at 480 nm), derived from S4C Fig, were plotted versus the relevant GdnCl concentrations. (D) The θ_280_ (ellipticity at 280 nm) values, extracted from S4D Fig, were normalized [43] and plotted versus the matching GdnCl concentrations.

Many proteins were unfolded by different mechanism when urea was replaced with GdnCl [43, 52–55]. To understand about the GdnCl-induced unfolding of SarA, we recorded the far-UV CD and the intrinsic Tyr fluorescence spectra of rSarA in the presence of 0–5 M GdnCl separately (S4A and S4B Fig). The ellipticity values of rSarA (at 222 nm), which were marginally increased upon increasing the GdnCl concentrations from ~0 to 0.5 M, remained nearly unchanged at ~0.5–1.5 M GdnCl (Fig 4A). The ellipticity values were, however, decreased ~90% when the GdnCl concentrations were raised from ~1.5 to 3 M. At ~3–5 M GdnCl, very little change of ellipticity values was noticed. Unlike the CD values, Tyr fluorescence intensity values were regularly enhanced upon increasing the GdnCl concentrations from ~0 to 1.75 M (Fig 4B). Thereafter, Tyr fluorescence intensity values were reduced gradually when the GdnCl concentrations were further increased to ~3.5 M. The fluorescence intensity values, however, tended to increase again at the GdnCl concentrations of >3.5 M. No remarkable change in the λ_max_ values was noticed in the presence of GdnCl. As demonstrated above, the exact reason for the increase or decrease of the Tyr fluorescence intensity of rSarA at different GdnCl concentrations is not clear. The unfolding curve of rSarA (generated using its Tyr fluorescence values) was, however, similar to that observed for the apple 4 domain of a coagulation factor [56]. Like this domain, rSarA most possibly lost its secondary and tertiary structures upon increasing the GdnCl concentrations from ~1.75 to 3 M. There was complete unfolding of rSarA at the GdnCl concentrations of >3M.

To find out as to whether the GdnCl-induced unfolding of rSarA was reversible in nature, we recorded the far-UV CD spectra of equimolar concentrations of native, unfolded, and refolded rSarA. S5A Fig showed that the spectrum of the refolded rSarA overlapped completely with that of native rSarA. A gel shift assay picture also revealed that the refolded rSarA bound to the ^32^P-labeled hla DNA considerably (S5B Fig). The results together suggest the complete refolding of rSarA that was unfolded with 5 M GdnCl. For reasons not known, the DNA binding affinity of refolded rSarA (prepared from either urea- or GdnCl-treated rSarA) was relatively higher than that of the native protein.

### Unfolding intermediates generated by GdnCl

The unfolding curves (mentioned in Fig 4A and 4B) resulted at 0–5 M GdnCl did not show fitting to a two- or three-state equation (data not shown) [24, 25], indicating the formation of some intermediates during the GdnCl-induced unfolding of rSarA. To prove the hypothesis, we investigated the GdnCl-induced unfolding of rSarA by ANS fluorescence (S4C Fig) and near-UV CD spectroscopy (S4D Fig). The ANS fluorescence intensity of rSarA that reached the highest level at ~0.5 M GdnCl, was decreased slowly when the GdnCl concentrations were raised from ~0.5 to 3 M (Fig 4C). There was no change of ANS fluorescence intensity upon further increasing the GdnCl concentrations to 4 M. The data suggest the formation of at least one rSarA intermediate that carries a maximum extent hydrophobic surface at ~0.5 M GdnCl. Fig 4D showed that the ellipticity values of rSarA at 280 nm were decreased by about 50% in the presence of 0.5–5 M GdnCl. The data suggest that the rSarA intermediate generated at ~0.5 M GdnCl might have a molten globule-like structure [38] as it carried significantly less tertiary but an excess secondary structure (Fig 4A) at this denaturant concentration.

**Fig 5:**
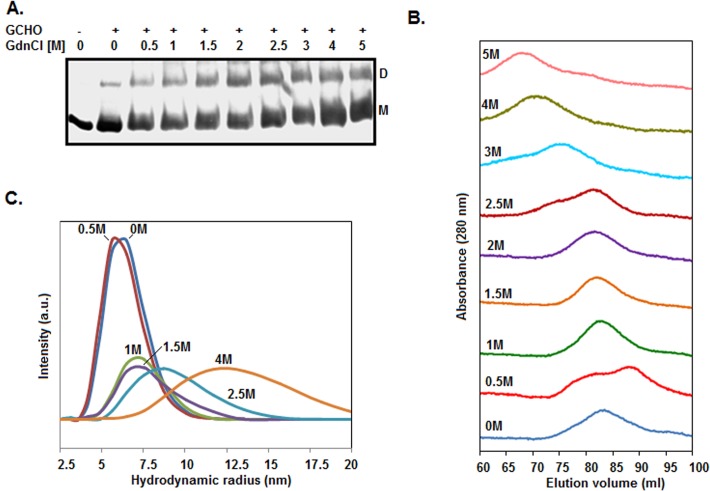
Size and shape of rSarA in the presence/absence of GdnCl. (A) Analysis of the chemically cross-linked rSarA molecules by SDS-13.5% PAGE. Samples containing rSarA were pre-equilibrated with 0–5 M GdnCl before treating them with glutaraldehyde (GCHO). D and M indicate dimer- and monomer-specific rSarA. (B) Gel filtration chromatography of rSarA at the indicated concentrations of GdnCl. (C) DLS study of rSarA at the indicated concentrations of GdnCl.

To discern as to whether the rSarA intermediate (formed at ~0.5 M GdnCl), like native rSarA, exists as a dimer in solution, we performed the glutaraldehyde-mediated cross-linking experiment of this protein in the presence of 0–5 M GdnCl. Unlike urea (Fig 3A), GdnCl did not dissociate the rSarA dimer even at the concentrations that unfolded it completely (Fig 5A). In addition, there was an increase of the intensity of rSarA-specific band from ~22 to ~42%, when GdnCl concentration was raised from 0–0.5 M to 2.5 M. No further increase of the intensity of rSarA dimer-specific band was noticed at the GdnCl concentration of >2.5 M. The existence of rSarA as a dimer at 0.5 M and higher GdnCl concentrations suggested that the dimeric form of rSarA (native/intermediate) underwent denaturation directly in the presence of this denaturant.

To verify the chemical cross-linking data, we studied the 0–5 M GdnCl-treated rSarA samples by gel filtration chromatography. All of the elution profiles are presented in Fig 5B. The 0.5 M GdnCl-equilibrated rSarA yielded two rough peaks, whereas, other samples produced primarily a single peak. The retention volumes of 0, 1, 1.5, 2, 2.5, 3, 4, and 5 M GdnCl-treated rSarA were 83.47, 81.6, 81.5, 81.25, 81.38, 75.13, 70.38, and 67.88 ml, respectively. In contrast, two peaks of 0.5 M GdnCl-exposed rSarA corresponded to the elution volumes of 87.33 and 81.2 ml, respectively. As demonstrated from the chromatographic data of the urea-treated rSarA (Fig 3C), the elution profiles of dimeric rSarA provided important hints about its shape in the presence of GdnCl. The earlier elution of rSarA at 3–5 M GdnCl might be due to its near complete to complete unfolded form. In contrast, a little swelling of dimeric rSarA at 1–2.5 M GdnCl might have led to its elution prior the native rSarA. The formation of an incomplete peak with the elution volume of ~75 ml at 2.5 M GdnCl indicates that a part of rSarA may exist as an unfolded form too at this denaturant concentration. The dimeric rSarA at 0.5 M GdnCl, unlike the dimeric rSarA at 1–2 M GdnCl, most likely remained as the two stable forms in the aqueous solution. One of the forms was eluted after the elution of GdnCl-unexposed rSarA, indicating that it possesses a relatively compact conformation. The compressed form, however, disappeared completely at 1 M and higher concentrations of GdnCl. Conversely, the shape of the second form appeared to be nearly identical to that of 1–2 M GdnCl-exposed rSarA, as they possessed similar elution volumes.

To confirm the gel filtration chromatography data, we performed DLS experiments of rSarA in the presence of 0–4 M GdnCl. All of the rSarA samples (pre-equilibrated/unequilibrated with GdnCl) showed a single peak with a distinct hydrodynamic radius (Fig 5C). At 0, 0.5, 1, 1.5, 2.5, and 4 M GdnCl, the apparent hydrodynamic radii of rSarA were ~6.50, ~5.62, ~7.53, ~7.53, ~8.72, and ~11.69 nm, respectively. The data mostly supported the gel filtration chromatography data and confirmed the formation of two intermediates during the unfolding of rSarA in the presence of GdnCl. While one of them is formed at ~0.5 M GdnCl, another is mostly populated at ~1–2 M GdnCl. Both intermediates might possess a molten globule-like form as they retained sufficient secondary structures, carried relatively higher extents of hydrophobic surface, but lost their tertiary structures mostly (Fig 4). Apparently, the molten globule-like structure produced at ~0.5 M GdnCl possessed a compact shape, whereas, that generated at ~1–2 M GdnCl carried a swelled shape. The swelling of rSarA intermediate possibly led to the exposure of additional Lys residues on its surface. The increased contents of rSarA dimer (Fig 5A) that we noticed at 1–2 M GdnCl may be due to these cross-linkable residues.

### Thermal unfolding of rSarA

**Fig 6:**
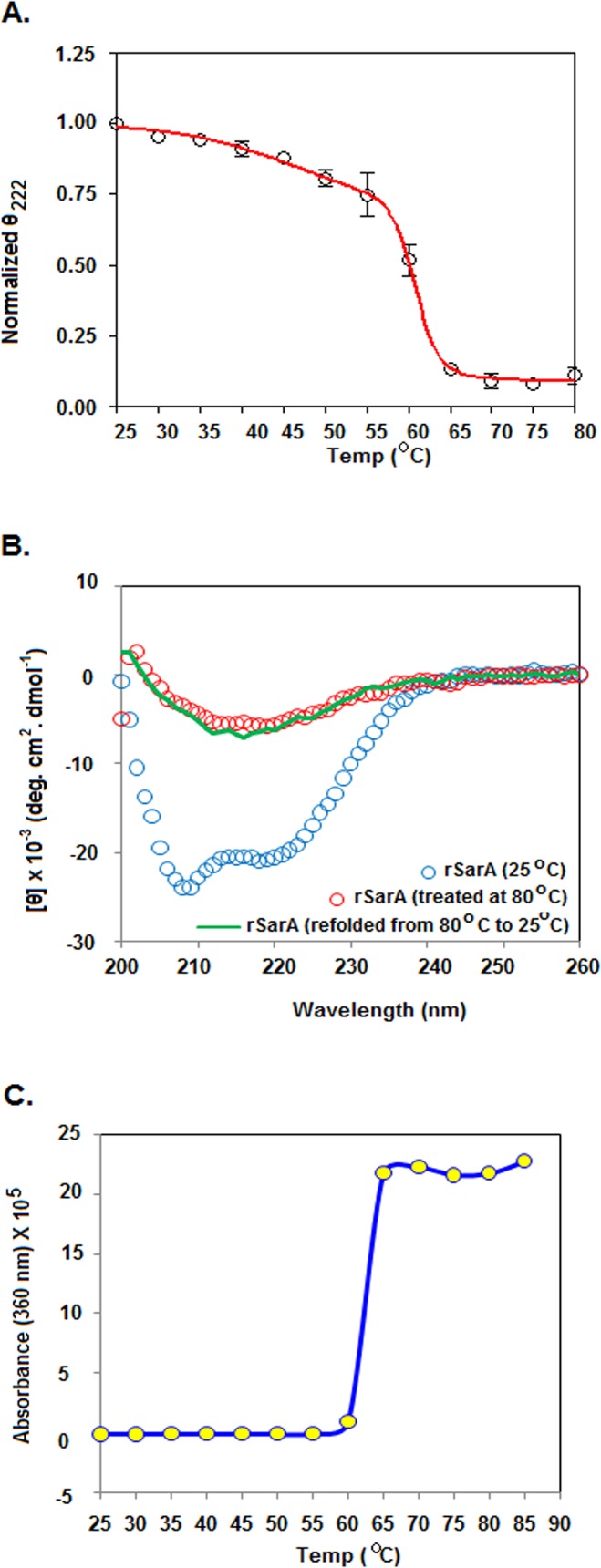
Thermal unfolding/refolding of rSarA by CD spectroscopy. (A) The plot of normalized θ_222_ (ellipticity at 222 nm) values versus temperature (Temp) was generated by a standard method [43] using the recorded CD data in S6 Fig The line through the ellipticity values is the best-fit curve. (B) Far-UV CD spectra of native, denatured, and refolded rSarA. (C) Thermal aggregation of rSarA by light scattering.

To find out the effects of temperature on rSarA, we recorded the far-UV CD spectra of this protein at 25°-80°C (S6 Fig). The plot of the negative ellipticity values of rSarA at 222 nm versus the temperature showed that there was ~25% decrease of the CD values upon increasing the temperature from 25° to 55°C (Fig 6A). Thereafter, the ellipticity values were reduced rapidly upon raising the temperature from 55° to 65°C. No further change of CD values was noticed at 65°-80°C, suggesting the complete unfolding of rSarA at 65°C and higher temperatures.

To test as to whether the thermal unfolding of rSarA is reversible in nature, we recorded the far-UV CD spectra of native, denatured (produced by incubating native rSarA at 80°C), and the refolded rSarA (made by slowly reducing the temperature of denatured rSarA to 25°C) as described above. Fig 6B showed that the CD spectrum of the refolded rSarA mostly coincided with that of the denatured rSarA, indicating that the denatured rSarA could not refold back to the native rSarA. rSarA also didn’t show any refolding when its temperature was slowly decreased from 60°/65° to 25°C (data not shown). The lack of refolding ability of rSarA was most likely due to the initiation of its aggregation at 60°C (Fig 6C). Taken together, we suggest that thermal unfolding of rSarA is irreversible in nature.

## Discussion

SarA, a global staphylococcal virulence regulator, has been studied extensively at the structural and functional levels [1, 2, 4–15, 17]. Our limited proteolysis data for the first time indicates that residues Leu 13, Asp 88, and Leu 108 in SarA are exposed in its surface since proteolysis occurs after/before them. The crystal structure of SarA shows that Leu 13 and Asp 88 are partially surface exposed, whereas, Leu 108 is buried completely within the structure of SarA [13]. Asp 88 is located in the loop region of the β-hairpin (a wing) in SarA [13]. Both this winged region (formed by residues 80 to 96) and the HTH motif (residues 53 to 77) of SarA are critical for its activity. In contrast, the N-terminal helices α1 (residues 9 to 28) and α2 (residues 34 to 45) and the C-terminal helix α5 (residues 99 to 121) are involved in the dimerization of SarA monomers [13, 19]. The cleavages at Leu 13 and Leu 108 are surprising as they are constituents of helices α1 and α5, respectively. The proteolytic cleavage in α-helix or β-sheet is, however, not unprecedented [27, 57].

The crystallographic *B*-value, another determinant of protein flexibility [36, 37], has been used by many researchers to explain their partial proteolysis data [27, 57]. By perusing the PDB file 2FRH, the *B*-values of residues 27 to 29, 47 to 105, and 120 to 124 were found significantly higher than those for other SarA residues [13]. The average *B*-values for Leu13, Asp 88, and Leu 108 are 32Å^2^, 146 Å^2^, and 47Å^2^ respectively [13]. As the mean *B*-value for SarA is 77.6Å^2^, Leu 13 and Leu 108 are relatively buried in the SarA dimer. The peptide bond formed by either Leu 108 and Ser 109 or Leu 13 and Ser 14 could be digested when SarA is in monomeric form. The relatively late appearance of fragments II or V (Fig 1B or 1C) partly supports the above hypothesis. We also found that the SarA fragment carrying Ala 2-Leu 108 was the only fragment whose theoretical molecular mass matched closely with the experimentally-determined mass of fragment V (Fig 1C and Table 1). Despite the cleavage at Leu 13, the N-terminal end of SarA may be relatively less flexible or exposed as no digestion occurred at (partially) exposed Phe 10 using chymotrypsin. Secondly, α5 possesses the highest fraction of surface-accessible residues. The average *B*-values for the first five residues at the N-terminal of helix α5 are also comparatively higher [13]. Collectively, the C-terminal half of SarA might be relatively more flexible in nature.

**Fig 7:**
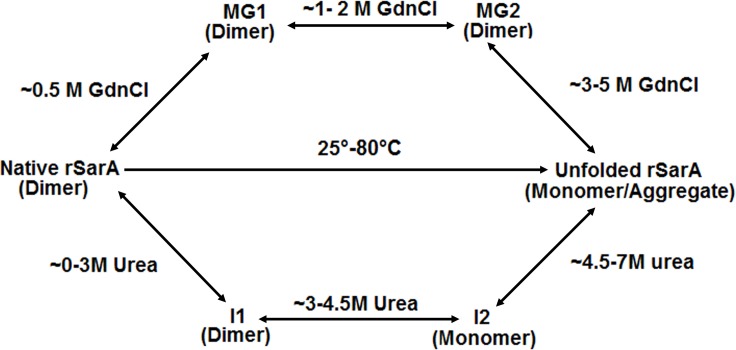
A schematic representation of the thermal and chemical-induced unfolding of rSarA. The MG1 and MG2 denote the putative rSarA molten globules those were generated at different GdnCl concentrations. The I1 and I2 indicate the urea-made rSarA intermediates.

We also demonstrated that rSarA was unfolded by different mechanisms in the presence of chemical and physical denaturants (Fig 7). While thermal unfolding pathway of rSarA was irreversible, unfolding pathway in the presence of either chemical denaturant was reversible along with the complete recovery of structure and function of rSarA. Despite reversibility, the unfolding of rSarA in the presence of either chemical denaturant was proceeded through the generation of at least two intermediates. All of the intermediates were formed at non-identical denaturant concentrations and possessed dissimilar properties. The rSarA intermediate (I1) that was formed at relatively lower urea concentrations existed primarily as the dimers like that of native rSarA. The secondary structure of I1 was also not perturbed notably. In contrast, its tertiary structure, shape and hydrophobic surface area were different from those of native rSarA. The other rSarA intermediate (I2), which was generated at comparatively higher urea concentrations, formed unfolded rSarA monomer when the concentrations of urea were raised from ~4.5 to 7 M. Unlike the native rSarA or I1, I2 existed predominantly as the monomers in the solution, possessed a relatively larger shape, contained very little secondary structure and was composed of a reduced extent of hydrophobic surface area. In comparison with I1, I2 also possessed a somewhat unfolded structure.

The shapes, surface hydrophobicity and Tyr fluorescence of two GdnCl-generated intermediates (namely, MG1 and MG2) did not match with each other (Fig 7). Both MG1 and MG2 were dimeric in solution, contained sufficient extents of secondary structure (which is a little higher or nearly similar to that of native rSarA) and populated at the GdnCl concentrations that did not initiate the unfolding of rSarA significantly. The dimeric rSarA intermediates were even little dissociated at the transition and post-transition regions, indicating that the mechanism of GdnCl-induced unfolding of rSarA is different from that of the urea-induced unfolding of this macromolecule. Unlike I1 and I2, both MG1 and MG2 were also suggested to possess a molten globule-like structure [38].

The way different denaturants (such as heat, urea, and GdnCl) unfold a protein is not known clearly [58–62]. These agents seemed to denature proteins mostly by disrupting their hydrogen bonds and hydrophobic interactions. Despite the similar mode of action, rSarA, like many other proteins [43, 52–55, 63–67], was unfolded by dissimilar mechanisms in the presence of the above denaturants. One of the reasons as to why the GdnCl-induced unfolding of proteins is different from the urea-induced or thermal unfolding could be the generation of Gdn^+^ ions from GdnCl (due to its dissociation in the aqueous solution) those weakened the electrostatic interactions in the proteins by neutralizing the (negative) charges on their surfaces. SarA is composed of twenty acidic and twenty-one basic amino acid residues. Analysis of the X-ray crystal structure of dimeric SarA with the Swiss-Pdb Viewer (at default setting) indicated that nearly 75% of the charged residues are located on the surface of SarA. The electrostatic charges of the charged residues on SarA surface would not have changed significantly at our working pH of 8.0. Most possibly, these surface-exposed charged residues play a key role in the maintenance of the stable conformation of SarA (in the aqueous solution), which was perturbed during its unfolding in the presence of Gdn^+^. Currently, it is not clear as to why thermal and urea-induced unfolding of rSarA proceeded by different mechanisms.

## Conclusions

The present investigations have not only verified the crystal structure of SarA but also provided clues about the folding-unfolding mechanism of this global staphylococcal regulator. The presence of a flexible region at the C-terminal end of the above single-domain protein was mostly supported by our partial proteolysis data with rSarA, a recombinant SarA. We have also stated that heat, urea, and GdnCl unfold rSarA by different ways. While thermal unfolding of rSarA led to its aggregation, the chemical-induced denaturation of this protein was completely reversible and occurred via the formation of two intermediates. The molecular properties of no intermediate, however, matched with those of other intermediates or the native rSarA. Unlike the urea-generated intermediates, both the GdnCl-made intermediates existed as the dimers in solution and possessed the characteristics of a molten globule. The unfolding data of rSarA could be useful in screening of the new anti-staphylococcal agents in the future.

## Supporting Information

S1 Fig Purification of functional rSarA. (A) Analysis of different protein containing fractions by SDS-13.5% PAGE. All fractions were prepared from SAU1311 cell extract. The uninduced, induced, supernatant, pellet, flow-thorough, wash, and elution fractions were loaded in lanes U, I, S, P, F, W, and E, respectively. Arrowhead indicates rSarA. Molecular masses of the marker (M) proteins (in kDa) were shown at the right side of the gel. (B) Western blotting analysis of rSarA. Arrowhead denoted rSarA that interacted with anti-his antibody. (C) Autoradiogram of the gel shift assay showing the binding of ^32^P-labeled hla DNA with varying concentrations of rSarA. (D) Plot of % hla DNA bound versus rSarA concentrations. Amounts of rSarA bound hla DNA were determined and plotted by a standard procedure as described in Materials and Methods.
(TIF)

S2 Fig Urea-induced unfolding of rSarA. (A) Far-UV CD spectra of rSarA in the presence of indicated concentrations of urea. (B) Intrinsic Tyr fluorescence spectra at 0–7 M urea.
(TIF)

S3 Fig Refolding of the urea-denatured rSarA. (A) Transverse urea-gradient gel electrophoresis of unfolded rSarA. (B) Far-UV CD spectra of unfolded, refolded, and native rSarA. (C) Gel shift assay of refolded rSarA using ^32^P-labeled hla DNA.
(TIF)

S4 Fig GdnCl-induced unfolding of rSarA. (A) Far-UV CD spectra of rSarA in the presence of indicated concentrations of GdnCl. (B) Intrinsic Tyr fluorescence spectra at 0–5 M GdnCl. (C) The ANS fluorescence spectra of rSarA in the presence of denoted concentrations of GdnCl. (D) Near-UV CD spectra of rSarA at 0–5 M GdnCl.
(TIF)

S5 Fig Refolding of the GdnCl-denatured rSarA. (A) Far-UV CD spectra of unfolded, refolded, and native rSarA. (B) Gel shift assay of refolded rSarA using ^32^P-labeled hla DNA. The intermediate band indicates contaminating band.
(TIF)

S6 Fig Thermal unfolding of rSarA. Far-UV CD spectra of rSarA at 25°-80°C.
(TIF)

## References

[1] Otto M. Basis of virulence in community-associated methicillin-resistant Staphylococcus aureus. Annu Rev Microbiol. 2010; 64:143–62. 10.1146/annurev.micro.112408.134309 20825344

[2] Plata K, Rosato AE, Wegrzyn G. Staphylococcus aureus as an infectious agent: overview of biochemistry and molecular genetics of its pathogenicity. Acta Biochim Pol. 2009; 56: 597–612. 20011685

[3] Stryjewski ME, Corey GR. New treatments for methicillin-resistant Staphylococcus aureus. Curr Opin Crit Care. 2009; 15: 403–12. 10.1097/MCC.0b013e32832f0a74 19561492

[4] Cheung AL, Bayer AS, Zhang G, Gresham H, Xiong YQ. Regulation of virulence determinants in vitro and in vivo in Staphylococcus aureus. FEMS Immunol Med Microbiol. 2004; 40: 1–9. 14734180 10.1016/S0928-8244(03)00309-2

[5] Bronner S, Monteil H, Prévost G. Regulation of virulence determinants in Staphylococcus aureus: complexity and applications. FEMS Microbiol Rev. 2004; 28: 183–200. 15109784 10.1016/j.femsre.2003.09.003

[6] Cue D, Lei MG, Lee CY. Genetic regulation of the intercellular adhesion locus in staphylococci. Front Cell Infect Microbiol. 2012; 2: 38 10.3389/fcimb.2012.00038 23061050 PMC3459252

[7] Arya R, Princy SA. An insight into pleiotropic regulators Agr and Sar: molecular probes paving the new way for antivirulent therapy. Future Microbiol. 2013; 8: 1339–53. 10.2217/fmb.13.92 24059923

[8] Didier JP, Cozzone AJ, Duclos B. Phosphorylation of the virulence regulator SarA modulates its ability to bind DNA in Staphylococcus aureus. FEMS Microbiol Lett. 2010; 306: 30–6. 10.1111/j.1574-6968.2010.01930.x 20337713

[9] Sun F, Ding Y, Ji Q, Liang Z, Deng X, Wong CC, et al Protein cysteine phosphorylation of SarA/MgrA family transcriptional regulators mediates bacterial virulence and antibiotic resistance. Proc Natl Acad Sci USA. 2012; 109: 15461–6. 22927394 10.1073/pnas.1205952109 PMC3458358

[10] Fujimoto DF, Higginbotham RH, Sterba KM, Maleki SJ, Segall AM, Smeltzer MS, et al Staphylococcus aureus SarA is a regulatory protein responsive to redox and pH that can support bacteriophage lambda integrase-mediated excision/recombination. Mol Microbiol. 2009; 74:1445–58. 10.1111/j.1365-2958.2009.06942.x 19919677 PMC2879156

[11] Morrison JM, Anderson KL, Beenken KE, Smeltzer MS, Dunman PM. The staphylococcal accessory regulator, SarA, is an RNA-binding protein that modulates the mRNA turnover properties of late-exponential and stationary phase Staphylococcus aureus cells. Front. Cell Infect Microbiol. 2012; 2: 26 10.3389/fcimb.2012.00026 22919618 PMC3417590

[12] Rechtin TM, Gillaspy AF, Schumacher MA, Brennan RG, Smeltzer MS, Hurlburt BK. Characterization of the SarA virulence gene regulator of Staphylococcus aureus. Mol Microbiol. 1999; 33: 307–316. 10411747 10.1046/j.1365-2958.1999.01474.x

[13] Liu Y, Manna AC, Pan CH, Kriksunov IA, Thiel DJ, Cheung AL, et al Structural and function analyses of the global regulatory protein SarA from Staphylococcus aureus. Proc Natl Acad Sci USA. 2006; 103: 2392–7. 16455801 10.1073/pnas.0510439103 PMC1413715

[14] Cheung AL, Nishina KA, Trotonda MP, Tamber S. The SarA protein family of Staphylococcus aureus. Int J Biochem Cell Biol. 2008; 40: 355–61. 18083623 10.1016/j.biocel.2007.10.032 PMC2274939

[15] Ballal A, Manna AC. Expression of the sarA family of genes in different strains of Staphylococcus aureus. Microbiology. 2009; 155: 2342–52. 10.1099/mic.0.027417-0 19389785 PMC2888119

[16] Chen PR, Bae T, Williams WA, Duguid EM, Rice PA, Schneewind O, et al An oxidation-sensing mechanism is used by the global regulator MgrA in Staphylococcus aureus. Nat Chem Biol. 2006; 2: 591–5. 16980961 10.1038/nchembio820

[17] Liu Y, Manna AC, Li R, Martin WE, Murphy RC, Cheung AL, et al Crystal structure of the SarR protein from Staphylococcus aureus. Proc Natl Acad Sci USA. 2001; 98: 6877–6882. 11381122 10.1073/pnas.121013398 PMC34446

[18] Poor CB, Chen PR, Duguid E, Rice PA, He C. Crystal structures of the reduced, sulfenic acid, and mixed disulfide forms of SarZ, a redox active global regulator in Staphylococcus aureus. J Biol Chem. 2009; 284: 23517–24. 10.1074/jbc.M109.015826 19586910 PMC2749125

[19] Zhu Y, Fan X, Zhang X, Jiang X, Niu L, Teng M, et al Structure of Rot, a global regulator of virulence genes in Staphylococcus aureus. Acta Crystallogr D Biol Crystallogr. 2014; 70: 2467–76. 10.1107/S1399004714015326 25195759

[20] Gordon CP, Williams P, Chan WC. Attenuating Staphylococcus aureus virulence gene regulation: a medicinal chemistry perspective. J Med Chem. 2013; 56: 1389–404. 10.1021/jm3014635 23294220 PMC3585718

[21] Sun F, Zhou L, Zhao B-C, Deng X, Cho H, Yi C, et al Targeting MgrA-mediated virulence regulation in Staphylococcus aureus. Chem Biol. 2011; 18: 1032–1041. 10.1016/j.chembiol.2011.05.014 21867918 PMC3163066

[22] Anfinsen CB. Principles that govern the folding of protein chains. Science. 1973; 181: 223–30. 4124164 10.1126/science.181.4096.223

[23] Pace CN. The stability of globular proteins. CRC Crit Rev Biochem. 1975; 3: 1–43. 238787 10.3109/10409237509102551

[24] Kim PS, Baldwin RL. Intermediates in the folding reactions of small proteins. Annu Rev Biochem. 1990; 59: 631–660. 2197986 10.1146/annurev.bi.59.070190.003215

[25] Pace CN, Shaw KL. Linear extrapolation method of analyzing solvent denaturation curves. Proteins Suppl. 2000; 4: 1–7. 10.1002/1097-0134(2000)41:4+<1::aid-prot10>3.3.co;2-u 11013396

[26] Righetti PG, Verzola B. Folding/unfolding/refolding of proteins: present methodologies in comparison with capillary zone electrophoresis. Electrophoresis. 2001; 22: 2359–74. 11519938 10.1002/1522-2683(200107)22:12<2359::AID-ELPS2359>3.0.CO;2-8

[27] Fontana A, de Laureto PP, Spolaore B, Frare E, Picotti P, Zambonin M. Probing protein structure by limited proteolysis. Acta Biochim Pol. 2004; 51: 299–321. 15218531

[28] Gianni S, Ivarsson Y, Jemth P, Brunori M, Travaglini-Allocatelli C. Identification and characterization of protein folding intermediates. Biophys Chem. 2007; 128: 105–13. 17498862 10.1016/j.bpc.2007.04.008

[29] Morris ER, Searle MS. Overview of protein folding mechanisms: experimental and theoretical approaches to probing energy landscapes. Curr Protoc Protein Sci. 2012; Chapter 28: Unit 28.2.1–22. 10.1002/0471140864.ps2802s68 22470128

[30] Kaplan J, DeGrado WF. De novo design of catalytic proteins. Proc Natl Acad Sci USA. 2004; 101: 11566–11570. 15292507 10.1073/pnas.0404387101 PMC511021

[31] Lassila JK, Privett HK, Allen BD, Mayo SL. Combinatorial methods for small-molecule placement in computational enzyme design. Proc Natl Acad Sci USA. 103: 2006; 16710–16715. 17075051 10.1073/pnas.0607691103 PMC1636520

[32] Radford SE. Protein folding: progress made and promises ahead. Trends Biochem Sci. 2000; 25: 611–618. 11116188 10.1016/s0968-0004(00)01707-2

[33] Waldron TT, Murphy KP. Stabilization of proteins by ligand binding: application to drug screening and determination of unfolding energetics. Biochemistry. 2003; 42: 5058–64. 12718549 10.1021/bi034212v

[34] Senisterra G, Chau I, Vedadi M. Thermal denaturation assays in chemical biology. Assay Drug Dev Technol. 2012; 10: 128–36. 10.1089/adt.2011.0390 22066913

[35] Mahendrarajah K, Dalby PA, Wilkinson B, Jackson SE, Main ER. A high-throughput fluorescence chemical denaturation assay as a general screen for protein-ligand binding. Anal Biochem. 2011; 411: 155–7. 10.1016/j.ab.2010.12.001 21138727

[36] Yuan Z, Zhao J, Wang ZX. Flexibility analysis of enzyme active sites by crystallographic temperature factors. Protein Eng. 2003; 16: 109–14. 12676979 10.1093/proeng/gzg014

[37] de Brevern AG, Bornot A, Craveur P, Etchebest C, Gelly JC. PredyFlexy: flexibility and local structure prediction from sequence. Nucleic Acids Res. 2012; 40: W317–22. 10.1093/nar/gks482 22689641 PMC3394303

[38] Arai M, Kuwajima K. Role of the molten globule state in protein folding. Adv Protein Chem. 2000; 53: 209–282. 10751946 10.1016/s0065-3233(00)53005-8

[39] Sambrook J, Russell DW. Molecular Cloning: A Laboratory Manual. 3rd edn. New York: Cold Spring Harbor Laboratory Press; 2001.

[40] Ganguly T, Das M, Bandhu A, Chanda PK, Jana B, Mondal R, et al Physicochemical properties and distinct DNA binding capacity of the repressor of temperate Staphylococcus aureus phage phi11. FEBS J. 2009; 276: 1975–85. 10.1111/j.1742-4658.2009.06924.x 19250317

[41] Ausubel FM, Brent R, Kingston RE, Moore DD, Seidman JG, et al Current Protocols in Molecular Biology. New Jersey: John Wiley & Sons, Inc.; 1998.

[42] Chien Y, Manna AC, Projan SJ, Cheung AL. SarA, a global regulator of virulence determinants in Staphylococcus aureus, binds to a conserved motif essential for *sar*-dependent gene regulation. J Biol Chem. 1999; 274: 37169–76. 10601279 10.1074/jbc.274.52.37169

[43] Jana B, Bandhu A, Mondal R, Biswas A, Sau K, Sau S. Domain structure and denaturation of a dimeric Mip-like peptidyl-prolyl cis-trans isomerase from Escherichia coli. Biochemistry. 2012; 51: 1223–37. 10.1021/bi2015037 22263615

[44] Biswas A, Mandal S, Sau S. The N-terminal domain of the repressor of Staphylococcus aureus phage Φ11 possesses an unusual dimerization ability and DNA binding affinity. PLoS One. 2014; 9: e95012 10.1371/journal.pone.0095012 24747758 PMC3991615

[45] Polley S, Jana B, Chakrabarti G, Sau S. Inhibitor-Induced Conformational Stabilization and Structural Alteration of a Mip-Like Peptidyl Prolyl cis-trans Isomerase and Its C-Terminal Domain. PLoS One. 2014; 9: e102891 10.1371/journal.pone.0102891 25072141 PMC4114562

[46] Creighton TE. Protein Structure: A Practical Approach. 2nd ed. New York: IRL Press at Oxford University Press; 1997.

[47] Lakowicz JR. Principles of fluorescence spectroscopy 2nd ed. New York: Kluwer Academic/Plenum; 1999.

[48] Goldenberg DP, Creighton TE. Gel electrophoresis in studies of protein conformation and folding. Anal Biochem. 1984; 138: 1–18. 6203436 10.1016/0003-2697(84)90761-9

[49] Banerjee V, Das KP. Interaction of silver nanoparticles with proteins: A characteristic protein concentration dependent profile of SPR signal. Colloids Surf B Biointerfaces. 2013; 111C: 71–79. 10.1016/j.colsurfb.2013.04.052 23792543

[50] Park YC, Bedouelle H. Dimeric tyrosyl-tRNA synthetase from Bacillus stearothermophilus unfolds through a monomeric intermediate. A quantitative analysis under equilibrium conditions. J Biol Chem. 1998; 273:18052–9. 9660761 10.1074/jbc.273.29.18052

[51] Cellini B, Bertoldi M, Montioli R, Laurents DV, Paiardini A, Voltattorni CB. Dimerization and folding processes of Treponema denticola cystalysin: the role of pyridoxal 5'-phosphate. Biochemistry. 2006; 45: 14140–54. 17115709 10.1021/bi061496l

[52] Liu CP, Li ZY, Huang GC, Perrett S, Zhou JM. Two distinct intermediates of trigger factor are populated during guanidine denaturation, Biochimie. 2005; 87: 1023–1031. 15927341 10.1016/j.biochi.2005.03.017

[53] Akhtar MS, Ahmad A, Bhakuni V. Guanidinium chloride- and urea-induced unfolding of the dimeric enzyme glucose oxidase, Biochemistry. 2002; 41: 3819–3827. 11888301 10.1021/bi0116700

[54] Rashid F, Sharma S, Bano B. Comparison of guanidine hydrochloride (GdnHCl) and urea denaturation on inactivation and unfolding of human placental cystatin (HPC) The Protein Journal. 2005; 24: 283–292. 16284726 10.1007/s10930-005-6749-5

[55] Singh AR, Joshi S, Arya R, Kayastha AM, Saxena JK. Guanidine hydrochloride and urea-induced unfolding of Brugia malayi hexokinase, Eur Biophys J. 2010; 39: 289–297. 10.1007/s00249-009-0539-5 19756573

[56] Riley PW, Cheng H, Samuel D, Roder H, Walsh PN. Dimer dissociation and unfolding mechanism of coagulation factor XI apple 4 domain: spectroscopic and mutational analysis. J Mol Biol. 2007; 367: 558–73. 17257616 10.1016/j.jmb.2006.12.066 PMC1945241

[57] Pal A, Chattopadhyaya R. Digestion of the lambda cI repressor with various serine proteases and correlation with its three dimensional structure. J Biomol Struct Dyn. 2008; 26: 339–54. 18808200 10.1080/07391102.2008.10507249

[58] Nandi PK, Robinson DR. Effects of urea and guanidine hydrochloride on peptide and non-polar groups. Biochemistry. 1984; 23: 6661–6668. 6529576 10.1021/bi00321a058

[59] Mayo SL, Baldwin RL. Guanidinium chloride induction of partial unfolding in amide proton exchange in RNase A. Science. 1993; 262: 873–6. 8235609 10.1126/science.8235609

[60] Monera OD, Kay CM, Hodges RS. Protein denaturation with guanidine hydrochloride or urea provides a different estimate of stability depending on the contributions of electrostatic interactions. Protein Sci. 1994; 3: 1984–91. 7703845 10.1002/pro.5560031110 PMC2142645

[61] Del Vecchio P, Graziano G, Granata V, Barone G, Mandrich L, Manco G, et al Temperature- and denaturant-induced unfolding of two thermophilic esterases. Biochemistry. 2002; 41: 1364–71. 11802739 10.1021/bi011146t

[62] Zou Q, Habermann-Rottinghaus SM, Murphy KP. Urea effects on protein stability: hydrogen bonding and the hydrophobic effect. Proteins. 1998; 31: 107–15. 9593185

[63] Shah MA, Mishra S, Chaudhuri TK. Structural stability and unfolding transition of β-glucosidases: a comparative investigation on isozymes from a thermo-tolerant yeast. Eur Biophys J. 2011; 40: 877–89. 10.1007/s00249-011-0706-3 21538058

[64] Kishore D, Kundu S, Kayastha AM. Thermal, chemical and pH induced denaturation of a multimeric β-galactosidase reveals multiple unfolding pathways. PLoS One. 2012; 7: e50380 10.1371/journal.pone.0050380 23185611 PMC3503960

[65] Pica A, Russo Krauss I, Castellano I, Rossi M, La Cara F, Graziano G, et al Exploring the unfolding mechanism of γ-glutamyltranspeptidases: the case of the thermophilic enzyme from Geobacillus thermodenitrificans. Biochim Biophys Acta. 2012; 1824: 571–7. 10.1016/j.bbapap.2012.01.014 22322192

[66] Merlino A, Russo Krauss I, Rossi B, Vergara A, De Vendittis A, Marco S, et al Identification of an active dimeric intermediate populated during the unfolding process of the cambialistic superoxide dismutase from Streptococcus mutans. Biochimie. 2012; 94: 768–75. 10.1016/j.biochi.2011.11.008 22155088

[67] Wang TF, Lin MG, Lo HF, Chi MC, Lin LL. Biophysical characterization of a recombinant aminopeptidase II from the thermophilic bacterium Bacillus stearothermophilus. J Biol Phys. 2014; 40: 25–40. 10.1007/s10867-013-9332-x 24165863 PMC3923962

