# Supplementary figures and images for: Chemical and Thermal Unfolding of a Global Staphylococcal Virulence Regulator with a Flexible C-Terminal End
Source: PLoS One. 2015 Mar 30;10(3):e0122168. doi: 10.1371/journal.pone.0122168 (PMC4379015; doi:10.1371/journal.pone.0122168)

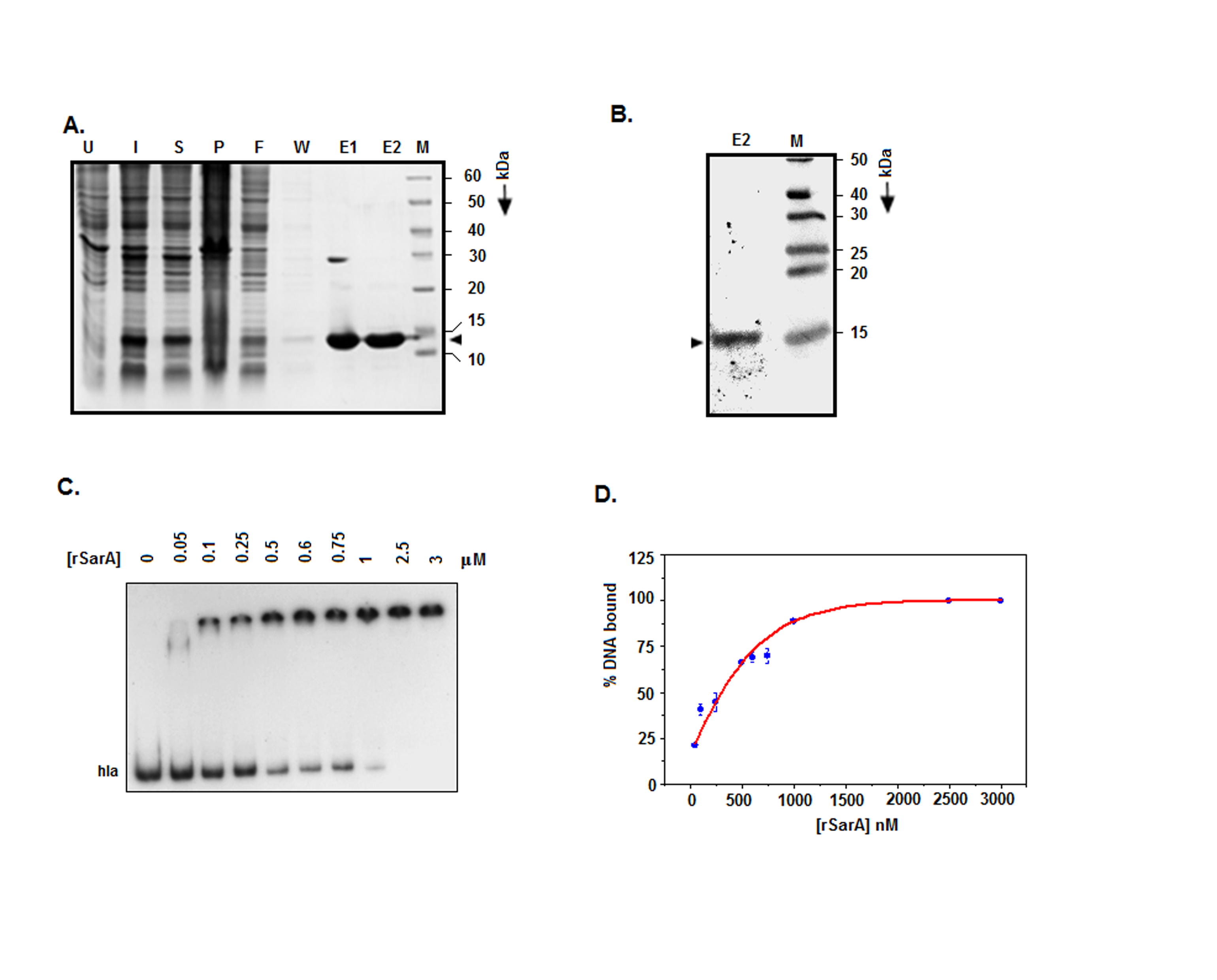

Supplement: S1 Fig — (A) Analysis of different protein containing fractions by SDS-13.5% PAGE. All fractions were prepared from SAU1311 cell extract. The uninduced, induced, supernatant, pellet, flow-thorough, wash, and elution fractions were loaded in lanes U, I, S, P, F, W, and E, respectively. Arrowhead indicates rSarA. Molecular masses of the marker (M) proteins (in kDa) were shown at the right side of the gel. (B) Western blotting analysis of rSarA. Arrowhead denoted rSarA that interacted with anti-his antibody. (C) Autoradiogram of the gel shift assay showing the binding of 32P-labeled hla DNA with varying concentrations of rSarA. (D) Plot of % hla DNA bound versus rSarA concentrations. Amounts of rSarA bound hla DNA were determined and plotted by a standard procedure as described in Materials and Methods. (TIF) [file pone.0122168.s001.tif]

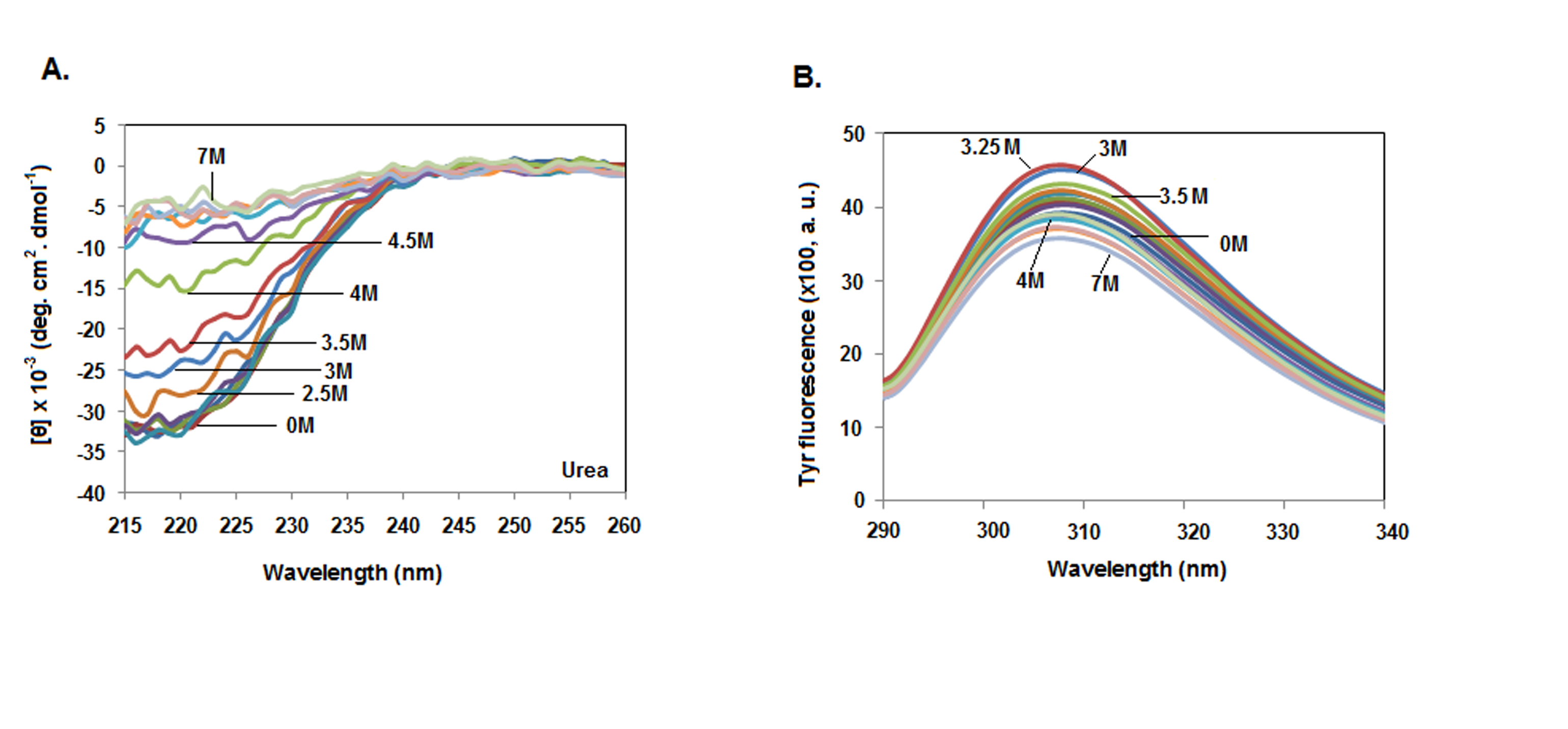

Supplement: S2 Fig — (A) Far-UV CD spectra of rSarA in the presence of indicated concentrations of urea. (B) Intrinsic Tyr fluorescence spectra at 0–7 M urea. (TIF) [file pone.0122168.s002.tif]

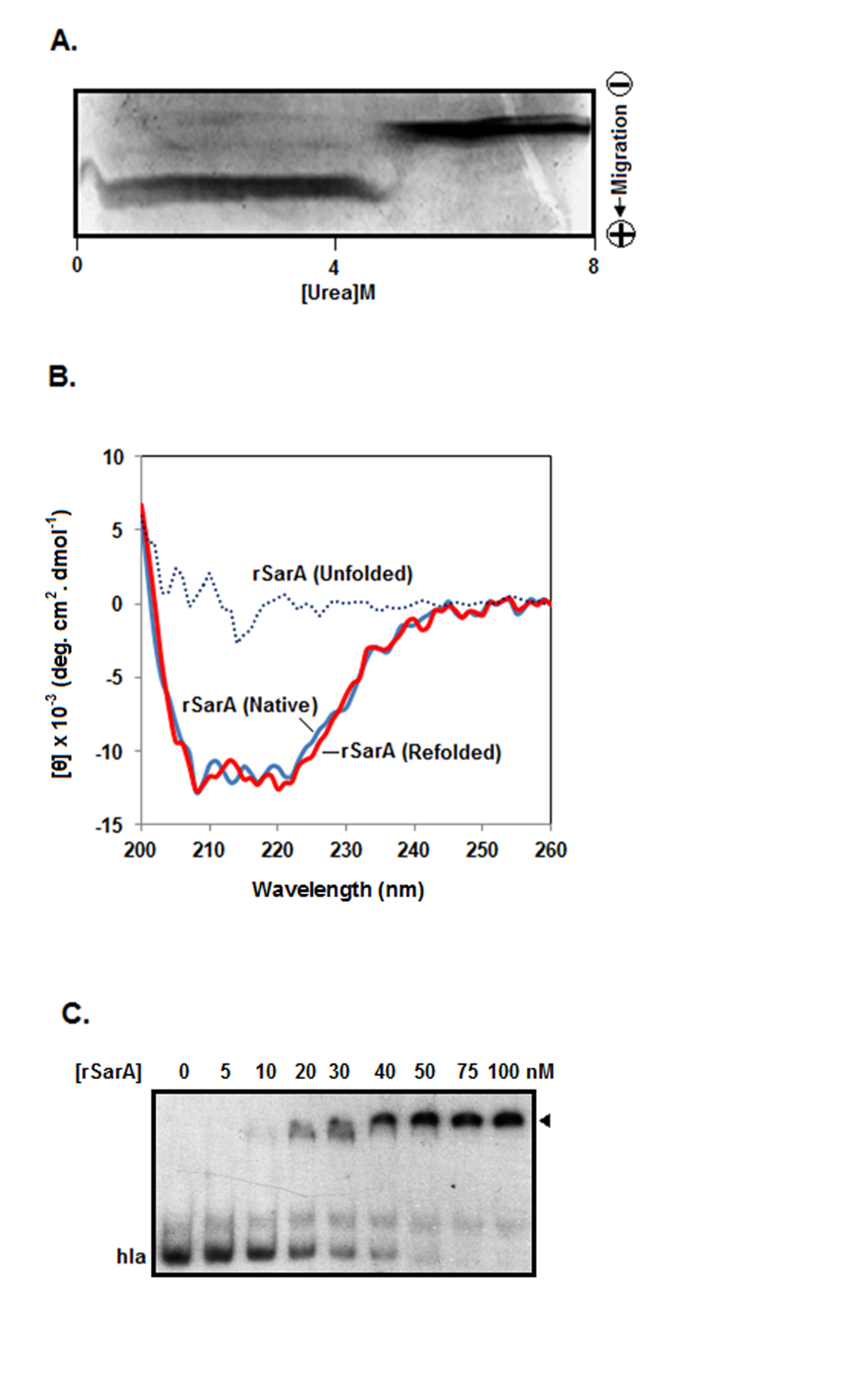

Supplement: S3 Fig — (A) Transverse urea-gradient gel electrophoresis of unfolded rSarA. (B) Far-UV CD spectra of unfolded, refolded, and native rSarA. (C) Gel shift assay of refolded rSarA using 32P-labeled hla DNA. (TIF) [file pone.0122168.s003.tif]

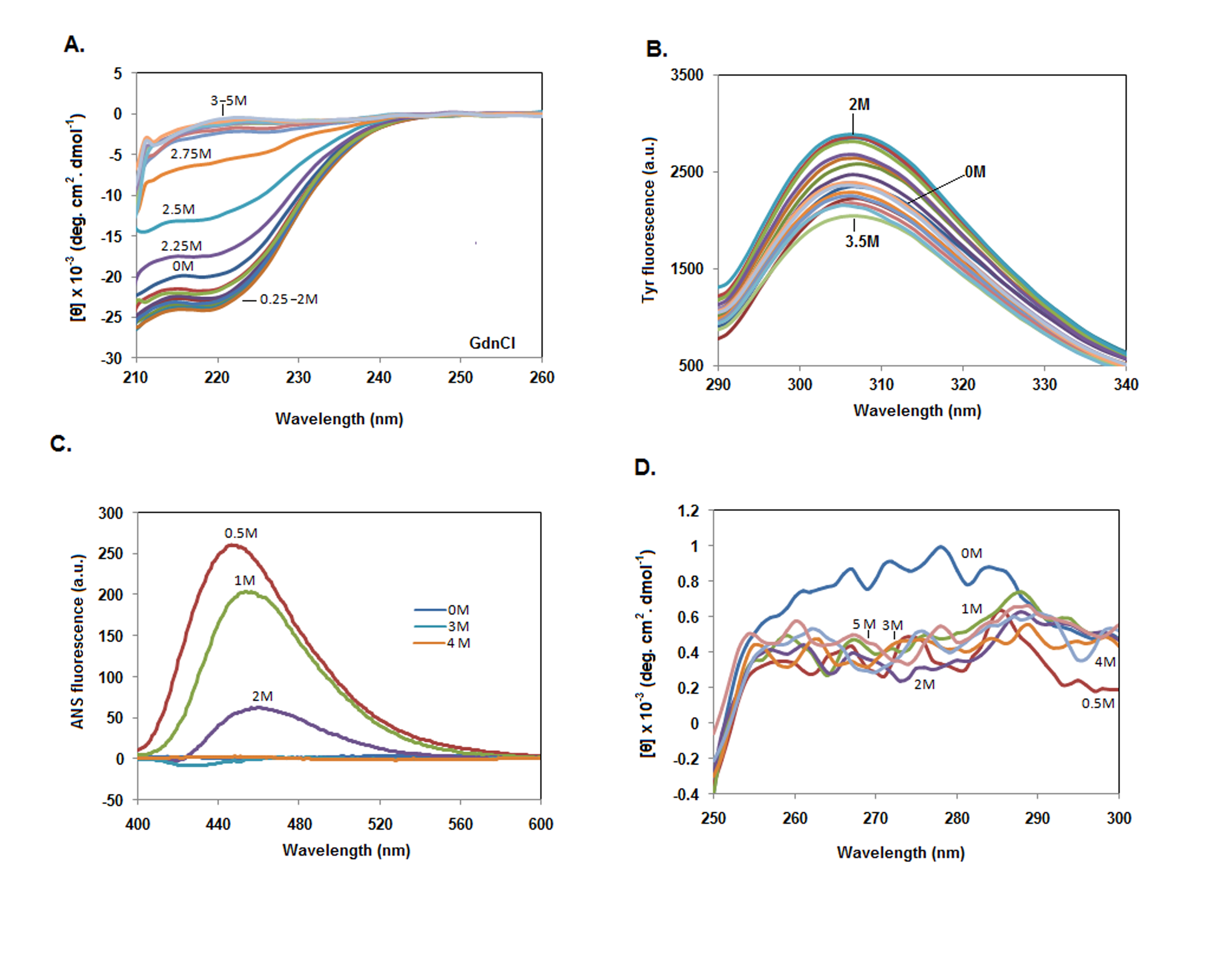

Supplement: S4 Fig — (A) Far-UV CD spectra of rSarA in the presence of indicated concentrations of GdnCl. (B) Intrinsic Tyr fluorescence spectra at 0–5 M GdnCl. (C) The ANS fluorescence spectra of rSarA in the presence of denoted concentrations of GdnCl. (D) Near-UV CD spectra of rSarA at 0–5 M GdnCl. (TIF) [file pone.0122168.s004.tif]

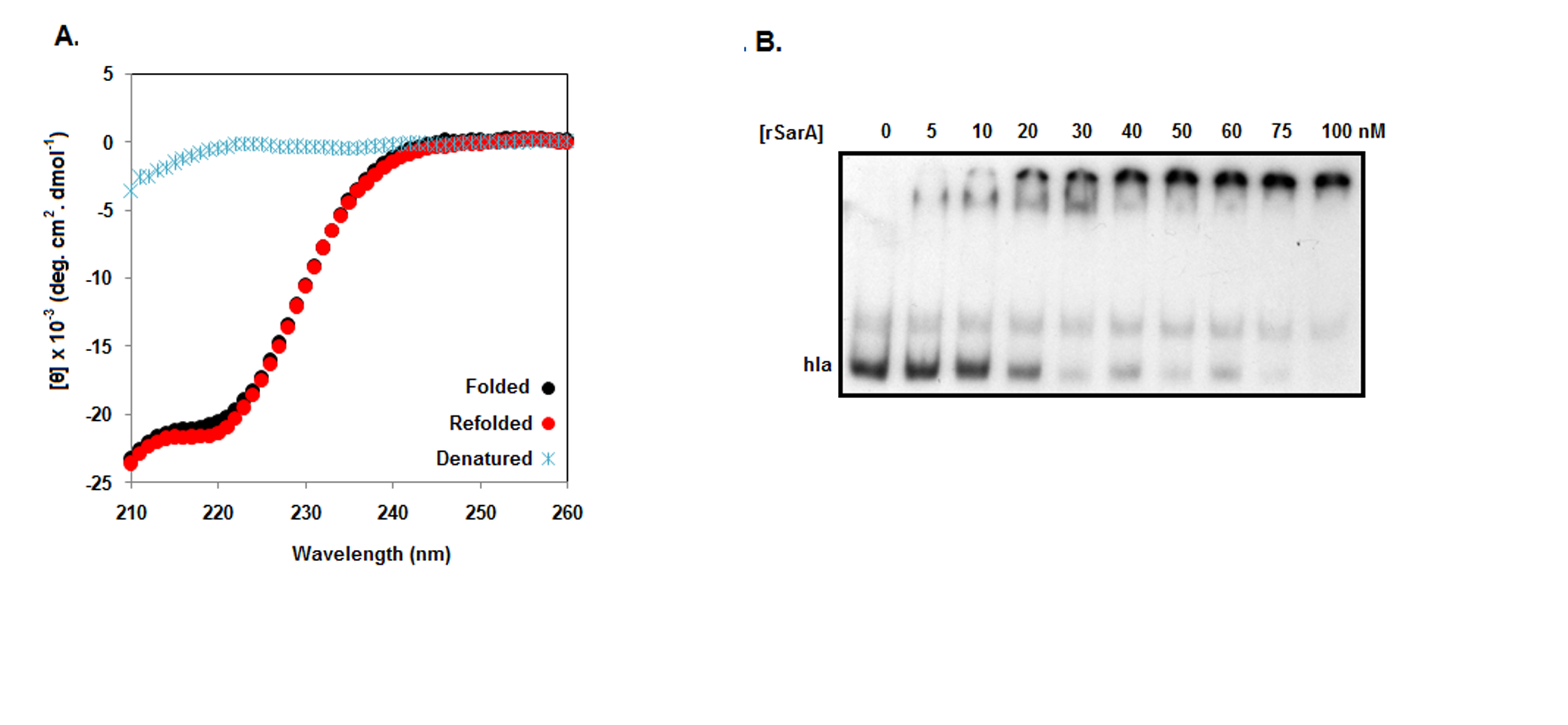

Supplement: S5 Fig — (A) Far-UV CD spectra of unfolded, refolded, and native rSarA. (B) Gel shift assay of refolded rSarA using 32P-labeled hla DNA. The intermediate band indicates contaminating band. (TIF) [file pone.0122168.s005.tif]

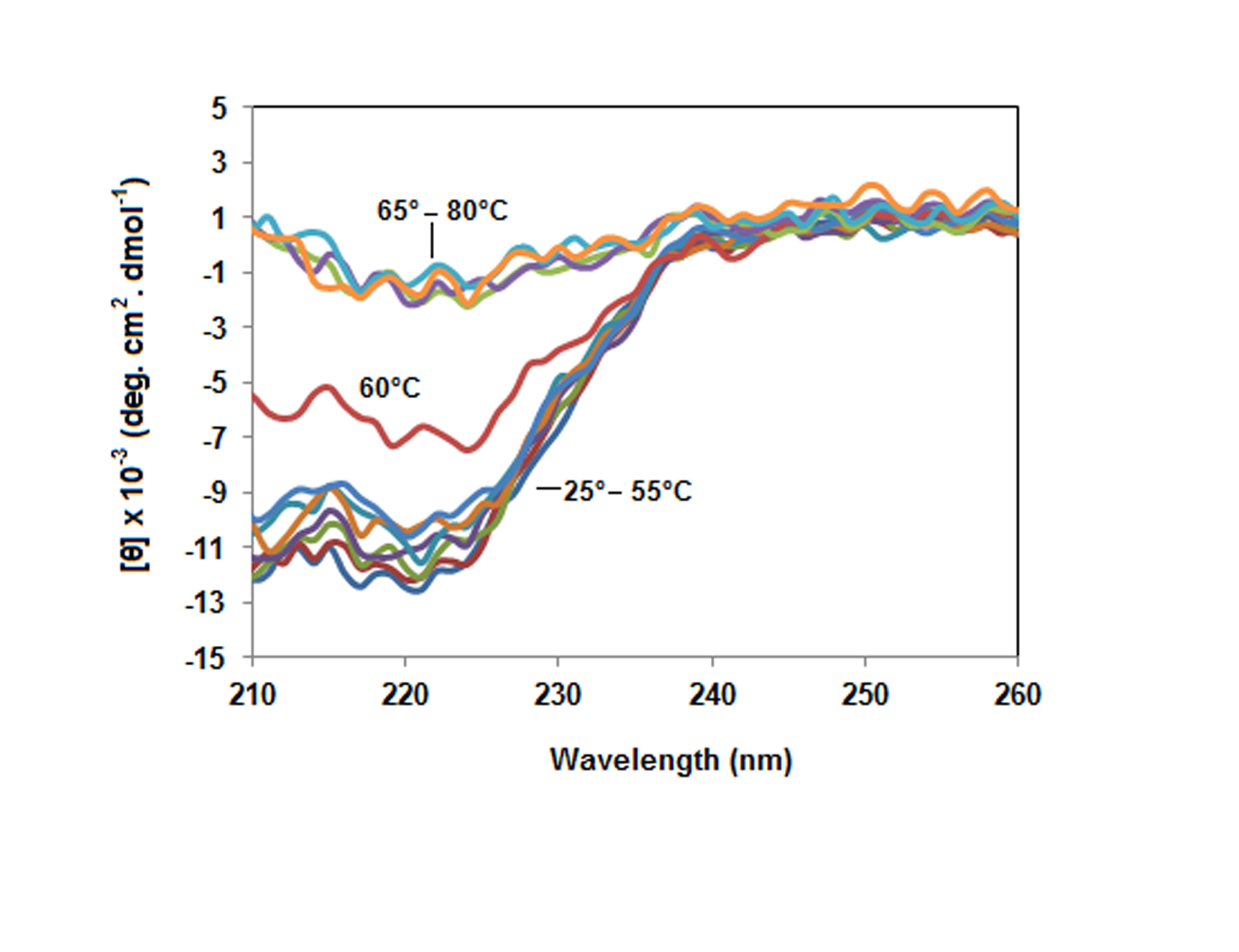

Supplement: S6 Fig — Far-UV CD spectra of rSarA at 25°-80°C. (TIF) [file pone.0122168.s006.tif]
